# The Edinburgh Medical and Surgical Journal, Exhibiting a Concise View of the Latest and Most Important Discoveries in Medicine, Pharmacy, and Surgery

**Published:** 1805-02-01

**Authors:** 


### CRITICAL ANALYSIS
### OF THE
### RECENT PUBLICATIONS
### ON THE
### DIFFERENT BRANCHES OF PHYSIC, SURGERY, AND MEDICAL PHILOSOPHY.

---

*The Edinburgh Medical and Surgical Journal, exhibiting a concise View of the latest and most important Discoveries in Medicine, Pharmacy, and Surgery. By a Society of Gentlemen of London and Edinburgh*, 1804.

By an advertisement prefixed to this number, we are informed that it is now to be offered to the public without the sanction of any particular names, on account of some new arrangements which have occurred since the work was first projected. The public, however, seems disposed to consider it as a continuation of the *Annals of Medicine* in a different, and, in our opinion, in a better form.

As we were in the habit of giving an analysis of the different articles in that work, we shall continue the same with the present.

The division, like our own, consists of original communications, analysis of books, and medical news. Of the former, the first article is the "History of Three Cases of Erythema Mercuriale, with Observations. By Thomas Spens, M. D. President of the Royal College of Physicians, and Physician to the Royal Infirmary of Edinburgh.

These cases are, in every respect, similar to those lately published by Mr. Alley, of Dublin, and Dr. Moriarty, of Roscommon. The former gives them the general name of the mercurial disease; the latter, *lepra mercurialis.* On account of the great desquamation, the latter term appears to us less objectionable than either of the other. This desquamation, or, from its depth and rapidity, as it may sometimes be called, this excoriation with exudation, peculiar fœtor, and in some instances, fever, are the peculiar character of a disease which seems altogether the consequence of idiosyncrasy. In the cases here related, exposure to cold was added to the exhibition of mercury. But this, the author remarks, is so common an occurrence, as to be considered an insufficient cause, without a peculiarity of constitution. In Dr. Moriarty's cases, we might add, an increased heat of the atmosphere, more commonly was noticed when the disease most prevailed. The complaint has certainly not hitherto been marked in the manner we trust it will be in future. Dr. Spens, indeed, gives Mr. Bell the credit of having noticed it in his Treatise; but Mr. Bell says nothing of desquamation, fœtid exudation, or fever. Moreover, he speaks of the complaint, as *not an uncommon effect* of mercury.

The second article is, " An Account of Two Cases of Tumours in the Pelvis growing out of the sacrosciatic Ligament, one of which terminated fatally, and the other was cured by extracting the Tumour through an Incision made into the Cavity of the Pelvis, [illegible]rough the Perinæum. By P. P. Drew, M. D. Fermoy, County of Cork."

This is a very important paper, inasmuch as it authorises an operation, which, but for the event, some people might have called rash. In the first case, the boldness of the undertaking deterred the surgeons from attempting it, apprehending that the tumour might be connected with the large blood vessels in the inside of the pelvis. The increase of the tumour at last produced a total interruption to the passages of the urethra and rectum; and during the absence of Dr. Drew, the patient died convulsed.

On making a free opening into the pelvis after death, the tumour was easily turned out, having no communicating blood vessels, and only a slight attachment to the surrounding parts, excepting at its neck, which seemed to grow out of the sacro-sciatic ligament. Its texture was gristly, and the body of the tumour was a fat gristly substance. This view of the parts after death, suggested to the operator a question, whether the tumour might not with safety have been removed during life, by making an incision on one side of the perinæum and anus, backwards towards the os coxygis?

About six months afterwards, the second case occurred. The first time Mr. Drew saw the woman, was the second day of her labour, in consultation with three other medical gentlemen, whose signatures he has thought it right to affix. The recollection of the former case, suggested the only remedy in the present. Either the Cæsarian operation, or the extraction of the tumour was absolutely necessary. Besides the well known danger of the former, even should it succeed, the diseased part would remain. Mr. Drew therefore undertook the operation of extracting the tumour by the perinæum, and succeeded. The woman was soon after delivered of a living child, and, when the case was transmitted, both were doing well. On the success of this important operation, which does so much honour to the operator and to surgery, it is unnecessary to make any remark; but we cannot dismiss the article without wishing gentlemen, who are most in the habit of deciding upon such cases, to consider whether some of those tumours, which arise from the ovaria, and are confined to the pelvis, might not with safety be extracted in this manner. We mean not to propose a hazardous operation, where the patient feels no other inconvenience than her increased bulk; but where the offices of the neighbouring parts are so much interrupted as to render life no longer desirable, such a proposal might be submitted to the patient.

Article the third, On the Treatment of Chorea Sancti Viti, by Purgatives. By John M'Mullin, Member of the Royal Medical Society of Edinburgh. The

The object of this paper is to show, that, admitting the disease in question to be a disease of debility, still purgatives may be necessary. This is what no prudent practitioner would doubt. Debility is for the most part rather an effect than a cause; and in our curative intention, the first business is to search for the cause. All Mr. M'Mullin's cases were in the Infirmary, probably among poor manufacturer's children, or others in a similar situation. Meagre diet, sedentary habits, and in some a period of life when we might expect chlorosis, probably produced a torpor in all the actions of the viscera, which a brisk purgative would be very likely to remove, and the diet and air of the hospital might be comparatively generous and pure, considering what such objects were accustomed to. The author remarks, that the fæces at the beginning of the treatment were, in every instance, black and fœtid. Fœtid is a very uncertain term for a stool, and the blackness might have been occasioned by the exhibition of chalybeates before the patients were admitted. However, these cases may be useful, if a general prejudice prevails that cathartics are never to be used in cases of chorœa.

The fourth article contains, "Two Cases in which diseased Portions of the Tongue were successfully removed by Means of Ligature. By Andrew Inglis, M. D. Fellow of the Royal College of Surgeons, and One of the Surgeons to the Royal Infirmary at Edinburgh." These cases are well reported.

The fifth article is a "Case of a remarkable Conformation of the Urinary and Genital Organs in a Female Child, (with an Engraving). By Mr. W. H. Coates, Surgeon to the Fifth Dragoon Guards. The subject was one of twins, a female, in all respects stout, notwithstanding her unhappy formation. The history may stand among the records of those unfortunates, but no practical advantage can be derived from it. As the author observes, it resembles pretty much the case given by Dr. Baillie, in the Medical and Chirurgical Transactions. We could wish Mr. C. to be a little more intelligible in his future reflections, should he offer any.

If the partiality of the public allowed a great discoverer to explain new ideas in new language, they may not be so well pleased with our author's "whimsicalities of Nature," and his conjectures whether chance or design might have produced some of these aberrations from the customary arrangement of parts. It is, however, but justice to add, that his only intention seems to institute an enquiry, how far the want of the anterior part of the bladder may render necessary, and be usually attended with those other alterations in the parts discoverable in this and many similar subjects. A well executed plate illustrates the description.

Article the sixth, "An Attempt towards a systematic Account of the Appearances connected with that Mal-formation of the urinary Organs, in which the Ureters, instead of terminating in a perfect Bladder, open externally on the Surface of the Abdomen.

By Andrew Duncan, Jun. M. D. Fellow of the Royal College of Physicians and Royal Society of Edinburgh."

What the former paper hints at, this promises very amply to supply. Dr. Duncan, with great industry and not less judgment, has collected all the cases related by different authors, in which the bladder was deficient, and endeavoured to make such an arrangement as may generalize, if not reduce to order this imperfect organization. After a few preliminary remarks, he divides his enquiries, by giving, first, an accurate account of a male subject to accompany Mr. Coates's description of a female. Secondly, a description of the urinary organs in all such subjects of both sexes, as have been recorded. Thirdly, other deviations from the natural structure, common to both sexes. Fourthly, a description of the genital organs of the male. Fifthly, a description of those organs in the female. Sixthly, general observations. The article in the present number, extends no further than to the fourth division. A reference to the male cases on record, follows. A plate, giving a description of a case which occurred to Mr. Astley Cooper, accompanies this article, which is to be concluded in the next number.

Article the seventh, " A Case of Catalepsy, with some Remarks on that Disease. By Richard Lubbock, M. D. of Norwich.

This curious disease, of which Dr. Cullen acknowledges he never saw *nisi simulatam*, has been accurately described by few modern authors. Dr. J. Jebb gave the history of a case, in which however many of his contemparies suspected he was deceived. Dr. Lubbock has seen several instances, two of which were in men. We are not disposed to question that gentleman's accuracy, nor do we doubt that the disease occurs much more frequently than is generally suspected, though the records are so few. The following are the particulars of this case in the author's own words.

" About twenty months ago, Mr. W——, of M——, the apothecary who attended X. Y. a lady who is the subject of this detail, wrote to me, and gave me the particulars of a complaint, appearing to him singular and anomalous, whieh has more or less continued to trouble her at intervals since that time. His account was, that X. Y. without any previous indisposition, was suddenly attacked, while at work or reading, with an appearance of insensibility, and loss of motion of the muscles, purely voluntary, for several minutes, although at the same time perfectly conscious of every thing passing around her; without any feeling of sickness or faintness, the colour of the countenance being unchanged, and in no degree different from what it was in the most perfect health. At that time there was no irregular or convulsive motion in any part of the body; and after remaining a short time motionless, and to appearance insensible, and at times without the power of speech, or of expressing her wants or wishes, by any kind of sign, she would in an instant resume her activity, without any any feeling of bodily or mental inconvenience from what had just taken place.

"During these attacks, the limbs were for the most part flexible; but now and then some degree of rigidity took place. At first, the attacks recurred but seldom, about once in a week; but afterwards they happened more frequently, and would equally take place whether the lady was walking or sitting. When the attack took place in the act of walking, she never injured herself by the fall, but, to use her own expression, she seemed to make a ridiculous display of ease and grace in falling."

From this account, Dr. Lubbock had no hesitation in pronouncing the disease catalepsy, and prescribed a variety of those remedies which are usually termed nervous and tonic, but with little success. The following is the account given of his first personal attendance.

"Upon my name being announced at my first visit, an occurrence of the disorder took place—she fell back upon the sofa, and remained motionless for some minutes, the pulse being undisturbed and regular, the colour of the countenance natural, and the respiration unaffected; and from this state she recovered in an instant, as if nothing had happened, and in full possession of the powers of body and mind. During the time she was under my immediate care, I frequently witnessed similar paroxysms when she was seated, and accompanied with rigidity of all, or of one or more of the extremities, and for a longer or shorter period, the limbs remaining in the position in which the seizure took place; and this position would immoveably remain the same, although, during the seizure, sleep should supervene: at times it happened, that the cataleptic state would come on during sleep. Thus, I have known one of the upper extremities caught nearly at right angles with the trunk of the body, at the instant of the attack, when X. Y. has been awake; and, sleep coming on during the seizure, the limb has retained the same position till the dissolution of the attack. I have also seen the complaint come on as she was walking across the room, when she would remain standing and motionless, with rigidity of the lower limbs for some time, but retaining frequently the use of the arms, and the power of speaking.

"I have seen the same rigidity suddenly affect the muscles of the head and neck, upon the head being turned towards the shoulder, to observe a person entering the room, in which position it would remain fixed for some time; and I have known the speech leave her entirely for forty-eight hours, although, during that time, she was able to walk three or four miles without fatigue. Indeed, the foregoing attacks scarcely ever prevented her from taking daily exercise, as, after they went off, she never felt any marked inability of exertion. And, to this list of irregularities of the influence of volition over the muscles, I may add, that at times she has been free from every other symptom of the attack, but the entire inability of shutting the eyelids; and at one time, for a few days,

 there there seems to have been something of the irregularity of the motion of the extremities that characterizes St. Vitus's Dance, but which soon went off."

After this, follows a further account of the remedies, which the author's candour induces him to speak of, without exalting their efficacy.

Nothing can be more perspicuous than the history of this disease, which is indeed not be wondered at, when we consider its author: but this consideration makes us regret the more that we cannot say as much for his reflexions.

"Many and curious," says our author, "are the speculations suggested to the mind, by reflecting on the phenomena of the present and similar cases. It may be questioned, whether catalepsy has been correctly arranged by Sauvages and Cullen, under the order *Comata* in the class *Neuroses.* There is one general fact to be collected from all such cases, which is, that the cause of these phenomena is intimately connected with that faculty of the mind termed *Volition.* And what is remarkable and meriting attention, the same kind and degree of action, whether of flexion or extension, that this power called volition is exerting over the voluntary muscles at the moment of the accession, is immoveably preserved till the termination of the attack. Thus the diffusive current of volition is, as it were, stopt or frozen up, and its course through the muscles becomes fixed, and remains precisely in the same state, till the spell that fetters its influence is dissolved, and the seizure goes off."

We cannot help thinking, that if Dr. Lubbock had made up his mind, with the same accuracy as he usually does before he offers his sentiments, he would have avoided a language well adapted to poetry. We, therefore, admire it in a Milton, and admit it in a Darwin. But we lament it in a philosopher, who at so early a period of life, and in so crude a state of the science, pointed to a revolution in chemistry, which was soon afterwards to follow.

The last original article is, "A Case of Enteritis, with Remarks. By Mr. James Rumsey, of Amersham, Member of the Royal College of Surgeons, of London, and of the Royal Society of Edinburgh. The only thing remarkable in this history is, that the author should think his practice new, or that the editors should, by a supplemental note, think it necessary to confirm it. In cases of severe pain in a belly, tense and tender to the touch, most practitioners would suspect inflammation, and bleed freely. The author observes, that constipation is a necessary effect of inflammation in the bowels, as vomiting is of gastritis. Admitting this to be the case, it does not follow, nor does either the author or editor hint that constipation always arises from such a cause. Where the symptoms of inflammation are present, as in the case before us, the practice is certainly judicious, and if thought new, it was at least well intended to enforce it.

The

The second part of the work consists of Critical Analysis of Books. Under the Third Division "Medical Intelligence," we have first a long and authentic account of the Surgical Academy, (Pepiniere) at Berlin. By D. Görcke, Surgeon General of the Prussian Army, and Director of the Academy. An Account of the Institution for the Cure and Prevention of Contagious Fever in this Metropolis; by Thomas Bateman, M. D. F. L. S. Physician to the Institution and to the Public Dispensary in Carey Street. Account of the Diseases in the Carey Street Dispensary from September 1 to November 30 inclusive. Two articles follow, which might be termed Communications; the first on the Anti-Variolous Powers of Vaccination; the second, on Pulmonary Tubercles. The last contains many useful hints on diseases which hitherto have not been discriminated with an accuracy at all proportionate to their frequent fatality.

